# Desmosome Assembly and Disassembly Are Membrane Raft-Dependent

**DOI:** 10.1371/journal.pone.0087809

**Published:** 2014-01-30

**Authors:** Sara N. Stahley, Masataka Saito, Victor Faundez, Michael Koval, Alexa L. Mattheyses, Andrew P. Kowalczyk

**Affiliations:** 1 Department of Cell Biology, Emory University School of Medicine, Atlanta, Georgia, United States of America; 2 Graduate Program in Biochemistry, Cell and Developmental Biology, Emory University School of Medicine, Atlanta, Georgia, United States of America; 3 Department of Dermatology, Emory University School of Medicine, Atlanta, Georgia, United States of America; 4 Division of Pulmonary, Allergy and Critical Care Medicine, Emory University School of Medicine, Atlanta, Georgia, United States of America; 5 Winship Cancer Institute, Emory University School of Medicine, Atlanta, Georgia, United States of America; University Medical Center Utrecht, Netherlands

## Abstract

Strong intercellular adhesion is critical for tissues that experience mechanical stress, such as the skin and heart. Desmosomes provide adhesive strength to tissues by anchoring desmosomal cadherins of neighboring cells to the intermediate filament cytoskeleton. Alterations in assembly and disassembly compromise desmosome function and may contribute to human diseases, such as the autoimmune skin blistering disease pemphigus vulgaris (PV). We previously demonstrated that PV auto-antibodies directed against the desmosomal cadherin desmoglein 3 (Dsg3) cause loss of adhesion by triggering membrane raft-mediated Dsg3 endocytosis. We hypothesized that raft membrane microdomains play a broader role in desmosome homeostasis by regulating the dynamics of desmosome assembly and disassembly. In human keratinocytes, Dsg3 is raft associated as determined by biochemical and super resolution immunofluorescence microscopy methods. Cholesterol depletion, which disrupts rafts, prevented desmosome assembly and adhesion, thus functionally linking rafts to desmosome formation. Interestingly, Dsg3 did not associate with rafts in cells lacking desmosomal proteins. Additionally, PV IgG-induced desmosome disassembly occurred by redistribution of Dsg3 into raft-containing endocytic membrane domains, resulting in cholesterol-dependent loss of adhesion. These findings demonstrate that membrane rafts are required for desmosome assembly and disassembly dynamics, suggesting therapeutic potential for raft targeting agents in desmosomal diseases such as PV.

## Introduction

The desmosome is an intercellular junction that mediates strong adhesion and anchors the intermediate filament cytoskeleton to the plasma membrane at sites of cell-cell contact [1], [2], [3]. Desmosomes are prominent in tissues that experience substantial mechanical stress, such as the skin and heart [4], [5]. Adhesive interactions in the desmosome are mediated by desmogleins and desmocollins, members of the cadherin superfamily of adhesion molecules [3]. Desmosomal plaque proteins, including plakoglobin and desmoplakin, tether the cytoplasmic tails of the desmosomal cadherins to the intermediate filament cytoskeleton. Plakophilins, a subgroup of the armadillo family, further cluster desmosomal cadherin complexes. This architectural arrangement integrates intercellular adhesive interactions and cytoskeletal elements, thereby mechanically coupling adjacent cells [1], [2], [6], [7]. Importantly, the function of both the desmosomal cadherins and the plaque proteins is essential for establishing and maintaining strong cell-cell adhesion, as evidenced by the numerous genetic, auto-immune, and infectious diseases that result when desmosomal protein function is compromised [8], [9], [10], [11], [12].

Although desmosomes mediate strong cell-cell adhesion, these structures are dynamic and exhibit tissue and differentiation specific changes in size and composition. The dynamics of desmosome assembly and disassembly must be precisely controlled to yield a junction both rigid enough to provide mechanical integrity to tissues, yet plastic enough to allow for remodeling during wound healing and development [13]. Alterations in desmosome assembly and disassembly are thought to compromise desmosome function in diseases such as the autoimmune blistering disease pemphigus vulgaris (PV) [13], [14], [15]. In PV, IgG auto-antibodies target the extracellular domain of the desmosomal cadherin desmoglein 3 (Dsg3), or both Dsg3 and Dsg1 [9], [11], [14], [16], [17], [18]. Histologically, the pemphigus family of diseases is characterized by the loss of adhesion, or acantholysis, between adjacent keratinocytes. Clinically, PV manifests as severe mucosal erosions as well as epidermal blisters [8], [9].

Recently, we and others have demonstrated that PV IgG aberrantly clusters cell surface Dsg3 [19], [20], leading to increased Dsg3 endocytosis and decreased steady state levels of Dsg3 at the plasma membrane [21], [22], resulting in desmosome disassembly. PV IgG-induced internalization occurs via a membrane raft-mediated pathway [23], indicating that Dsg3 raft association provides a means for desmosome regulation. Also known as lipid rafts or detergent resistant membranes (DRMs), membrane rafts (here, simply referred to as rafts) are highly ordered microdomains within the plasma membrane enriched in cholesterol and sphingolipids [24], [25]. Individual raft domains contain a small subset of select proteins and float freely within the membrane, but can cluster to form larger, ordered domains that function as platforms for a variety of cellular processes, such as signaling, endocytosis and membrane organization [25], [26]. Therefore, we speculated that rafts regulate the dynamics of desmosome assembly and disassembly, and thereby modulate normal keratinocyte adhesion, as well as keratinocyte responses to PV IgG. Indeed, several recent studies have demonstrated that desmosomal proteins, including Dsg2, Dsc2, plakoglobin and desmoplakin are raft associated [27], [28], [29]. Furthermore, classical preparations of desmosomes isolated from bovine snout are enriched in cholesterol and sphingolipids, providing further evidence of a tight association of desmosomes with membrane raft components [30], [31].

In the current study, we sought to determine if the PV antigen Dsg3 is also raft associated and if rafts play a functional role in regulating desmosomal adhesion. Using primary human keratinocytes, we demonstrate that Dsg3 is raft associated biochemically and colocalizes with raft markers as assessed by super resolution immunofluorescence microscopy. Disruption of membrane rafts via cholesterol depletion prevents desmosome assembly in response to increased extracellular calcium, thus establishing a role for rafts as critical regulators of desmosome formation. Interestingly, Dsg3 did not partition to rafts in cells lacking desmosomal proteins. Furthermore, in response to PV IgG, cell surface Dsg3 reorganizes into linear arrays, membrane projections that extend perpendicular from cell-cell borders. Super resolution immunofluorescence microscopy revealed that these linear arrays, which we have previously found to be active sites for Dsg3 endocytosis [32], are highly enriched in raft markers. Importantly, raft disruption prevents linear array formation, desmosome disassembly and the loss of cell adhesion in PV IgG treated cells. These results support a model in which membrane raft microdomains serve as a critical platform for the regulation of both desmosome assembly and disassembly.

## Results

### Dsg3 and Other Desmosomal Proteins are Membrane Raft Associated

**Figure 1:**
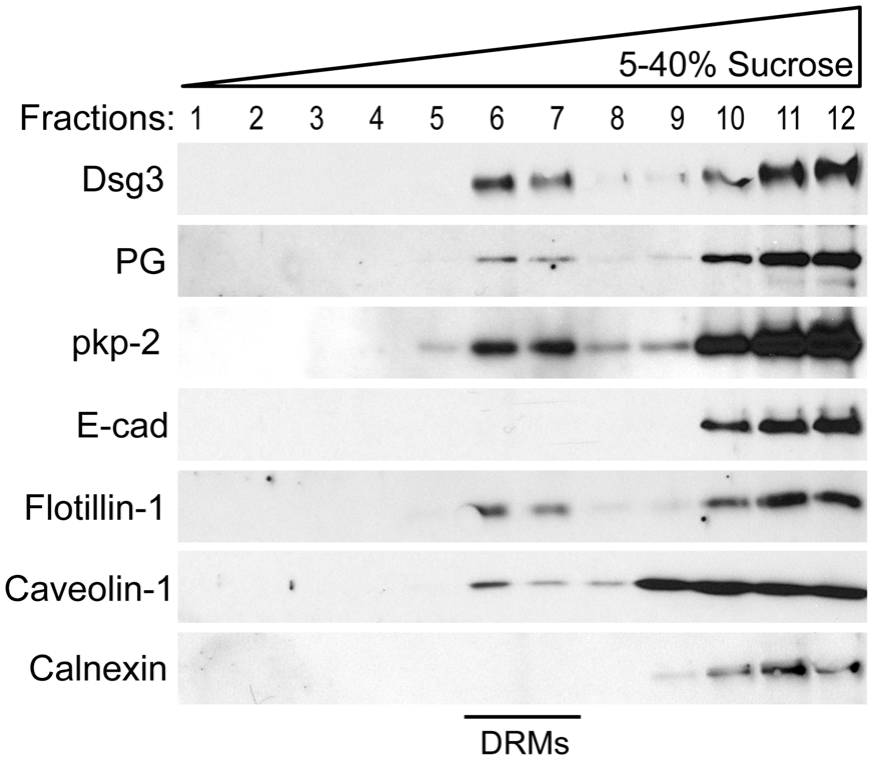
Dsg3 and other desmosomal proteins are membrane raft associated. Primary human keratinocytes were grown to confluence and switched to high calcium media for 16–18 hrs. Following detergent extraction (1% Triton X-100) and ultra-centrifugation on a 5–40% sucrose gradient, 12 fractions were sequentially removed from the gradient and processed via western blot. Dsg3 partitions to the buoyant raft fractions (DRMs, detergent resistant membranes) as indicated by the positive controls flotillin-1 and caveolin-1, and negative control calnexin. Desmosomal components plakoglobin (PG) and plakophilin 2 (pkp-2) were also found to be raft associated. E-cadherin, a classical cadherin of adherens junctions, is not enriched in membrane rafts.

**Figure 2:**
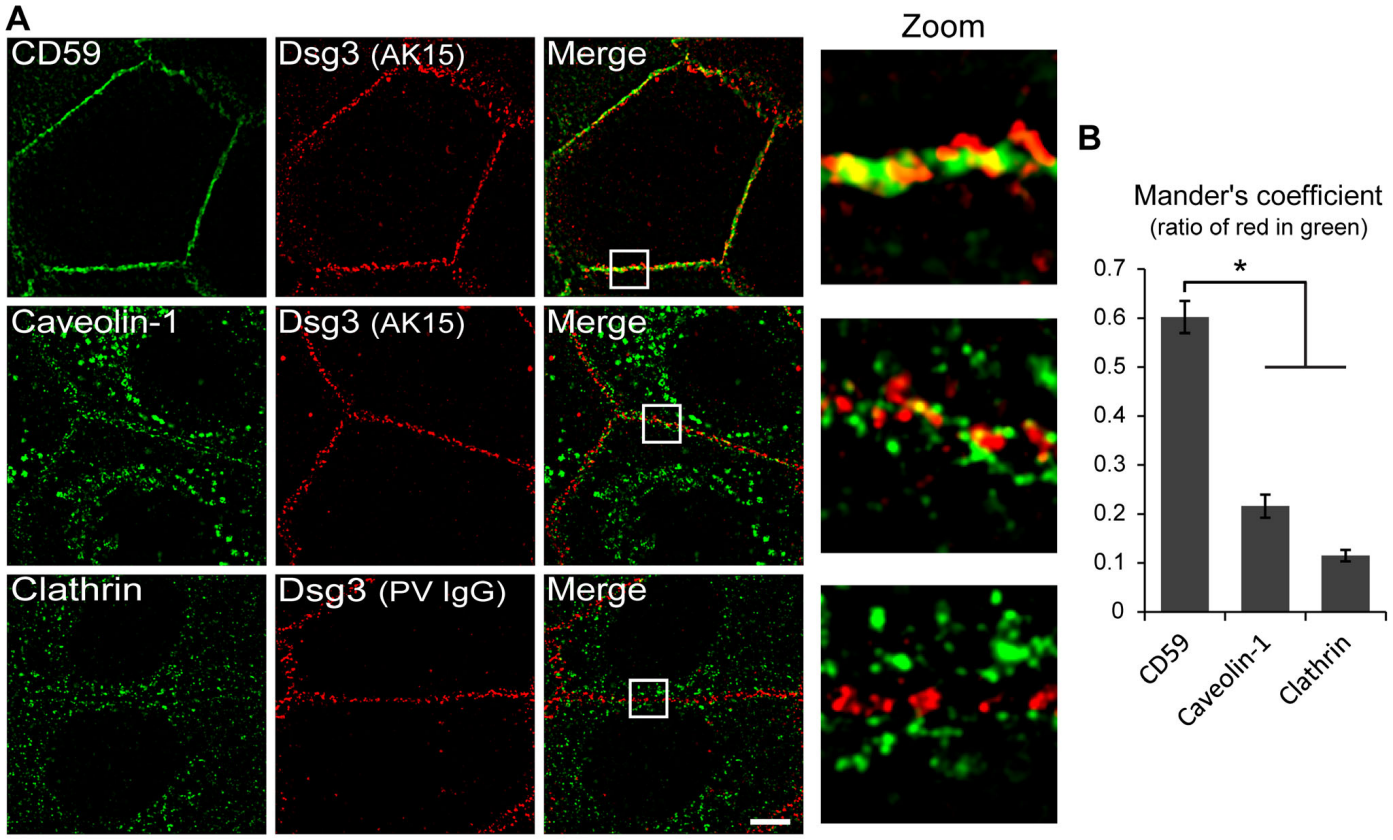
Dsg3 colocalizes with raft markers at cell-cell borders. (**A**) After switching human keratinocytes to high calcium media for 16–18 hrs, surface Dsg3 was labeled live for 10 min with Alexa Fluor 555-conjugated AK15 mAb (top 2 rows) or PV IgG. Dsg3 colocalization with raft markers CD59 (a GPI-anchored protein) and caveolin-1 were compared to colocalization with clathrin, a non-raft membrane component using SIM. Dsg3 colocalized substantially with CD59, moderately with caveolin-1 and very weakly with clathrin. (**B**) Quantification of Dsg3 colocalization. Mander’s coefficient was used to define the ratio of red fluorescence (Dsg3) found within green (CD59, caveolin-1 or clathrin). Means ± SEM (*n* = 20–37 border regions); *p<0.05. Scale bar in A, 5 µm.

To assess desmosomal protein association with membrane rafts, detergent resistant membranes (DRMs) were isolated from primary human keratinocytes. Following extraction in cold Triton X-100 and ultracentrifugation, buoyant DRMs and associated proteins partition to a characteristic density (∼25%) on a sucrose gradient [34]. Western blot analysis confirmed Dsg3 raft association with DRMs (Fig. 1, fractions 6 and 7) as identified by raft markers flotillin-1 and caveolin-1, and non-raft marker calnexin. Additionally, desmosomal proteins plakoglobin (PG) and plakophilin 2 (pkp-2) were found to be raft associated. Desmocollin 3 and desmoplakin also displayed partitioning to raft fractions (not shown). E-cadherin, a classical cadherin found in adherens junctions, failed to partition to rafts, demonstrating specificity for an enrichment of desmosomal components in rafts. Super resolution structured illumination microscopy (SIM) was used to determine if Dsg3 colocalized with raft markers at cell-cell borders. CD59, a GPI-anchored protein, and caveolin-1 (Cav-1) are proteins known to localize to membrane rafts and are commonly used as raft markers [29], [36]. Dsg3 was found to colocalize with both CD59 and caveolin-1, although to a much greater extent with CD59 (Fig. 2), suggesting a potential specificity for Dsg3 in CD59 containing raft domains. In contrast, Dsg3 did not colocalize with the non-raft marker clathrin. Together, these results suggest that raft association of desmosomal proteins is an integral aspect of desmosome regulation.

### Desmosome Assembly and Adhesion are Cholesterol Dependent

**Figure 3:**
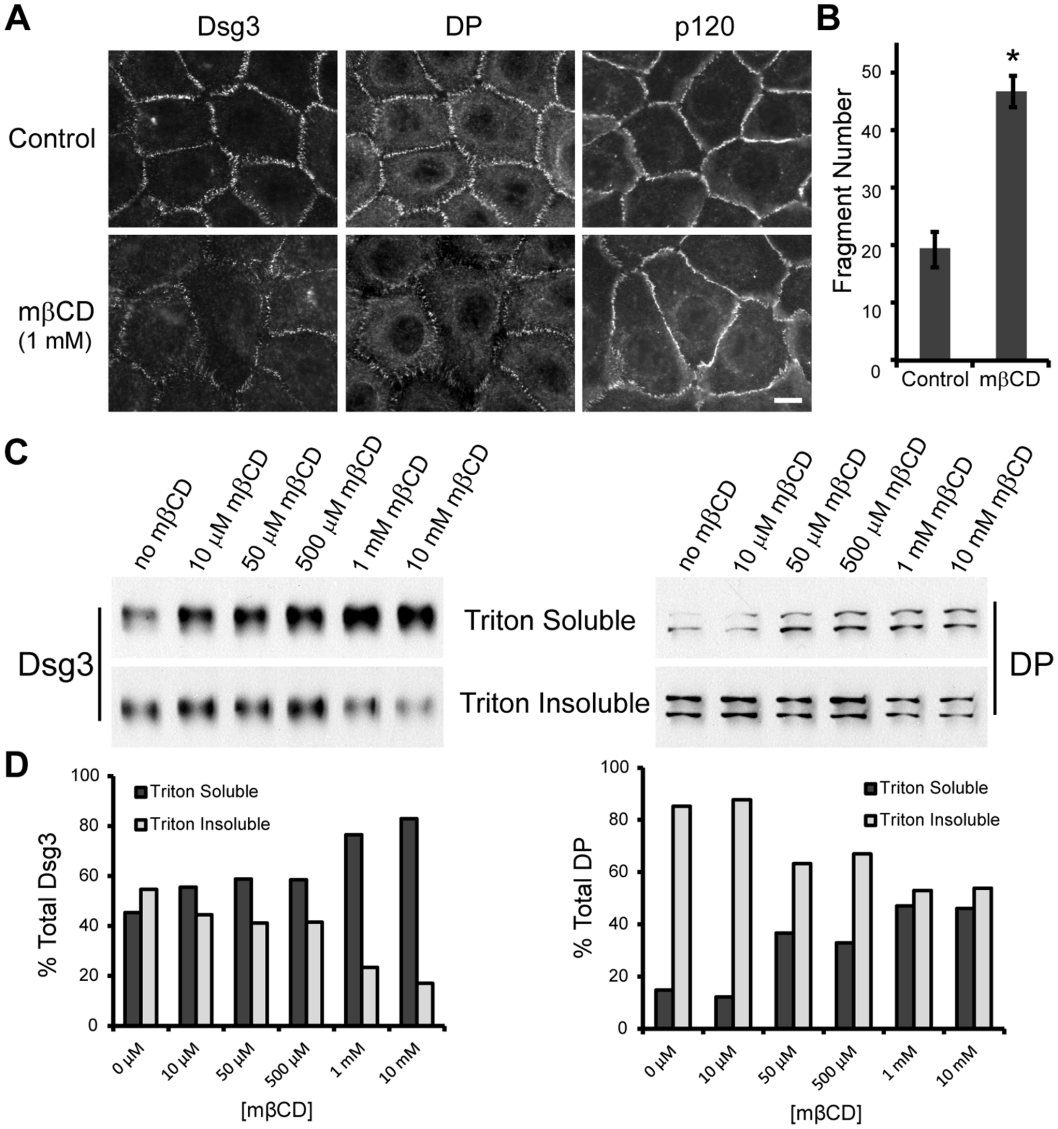
Desmosome assembly and adhesion are cholesterol dependent. (**A**) Human keratinocytes were treated with 1 mM mβCD (methyl-β-cyclodextrin) during a 3 hr switch from 50 µM to 550 µM calcium. Dsg3 was detected by labeling cells live with AK15 mAb during the last 15 min of the calcium switch. Under control conditions (no mβCD), Dsg3 and DP are recruited to cell borders. Border staining of both Dsg3 and DP is dramatically reduced in cells treated with mβCD, while border staining of adherens junction protein p120 remained similar to control. (**B**) Dispase-based fragmentation assay after keratinocytes were switched from a 50 µM to 550 µM calcium either in the absence or presence of 1 mM mβCD. Cells switched in the presence of mβCD showed a significant increase in the amount of fragmentation relative to control (no mβCD). Means ± SEM (*n* = 8 monolayers per group); *p<0.05. (**C**) Keratinocytes treated with varying concentrations of mβCD during a 6 hr switch from 50 µM to 550 µM were processed by sequential detergent extraction with 1% Triton X-100 and western blot to distinguish between the non-desmosomal and desmosomal pools of Dsg3 and DP. mβCD treatment caused a dose dependent shift of both Dsg3 and DP from Triton insoluble (desmosomal) to soluble (non-desmosomal) pool. (**D**) Quantification of Dsg3 and DP solubility changes in response to increasing mβCD concentrations. Scale bar in A, 10 µm.

To test if desmosome assembly was raft dependent, human keratinocytes were treated with methyl β-cyclodextrin (mβCD) during a low to high calcium switch to induce desmosome formation. Cholesterol depletion with mβCD is widely used as a method to disrupt membrane rafts [34], [37], [38]. Many cell types, including keratinocytes [37], [39], remain viable when treated with mβCD doses as high as 20 mM. However, at these doses we observed extensive cell rounding and shape changes (not shown) suggesting non-specific effects not directly attributable to cholesterol depletion. Additionally, high doses (≥10 mM) of mβCD exposure lead to cholesterol depletion from both raft and non-raft regions of the membrane, whereas lower doses have been shown to preferentially remove cholesterol from raft membrane domains [37]. Therefore, we treated cells with 1 mM mβCD (Fig. 3), which did not cause the cell shape changes observed with higher mβCD concentrations. Accumulation of both Dsg3 and DP at cell borders was reduced in cells treated with mβCD (Fig. 3A), suggesting that mβCD treatment prevented desmosome assembly. In contrast, border localization of adherens junction protein p120 was largely unchanged with mβCD treatment. A monolayer fragmentation assay was performed to confirm that mβCD treatment weakened adhesive strength [40]. Briefly, confluent keratinocyte monolayers were lifted off the culture dish with the enzyme dispase and then subjected to mechanical stress via pipetting. In this assay, increased monolayer fragmentation is indicative of weakened adhesion. Relative to control, cells switched to high calcium in the presence of mβCD showed a significant increase in fragmentation (Fig. 3B). Collectively, these findings indicate that raft disruption prevents desmosome assembly and weakens keratinocyte cell-cell adhesion strength.

To further test the effect of cholesterol depletion on the process of desmosome assembly, human keratinocytes were treated with varying concentrations of mβCD during a low to high calcium switch and then processed by sequential detergent extraction and western blot to distinguish between the non-desmosomal (Triton soluble) and desmosomal (Triton insoluble) pools of DP and Dsg3. The amounts of desmoplakin and Dsg3 in the insoluble or desmosomal pool are an indication of the relative assembly state of desmosomes. Treatment with mβCD caused a dose dependent shift of both desmoplakin and Dsg3 from the Triton insoluble to soluble pool (Fig. 3C, D). These results further indicate that desmosome assembly and raft association are intimately coupled.

### Dsg3 Raft Partitioning is Associated with Desmosome Assembly

**Figure 4:**
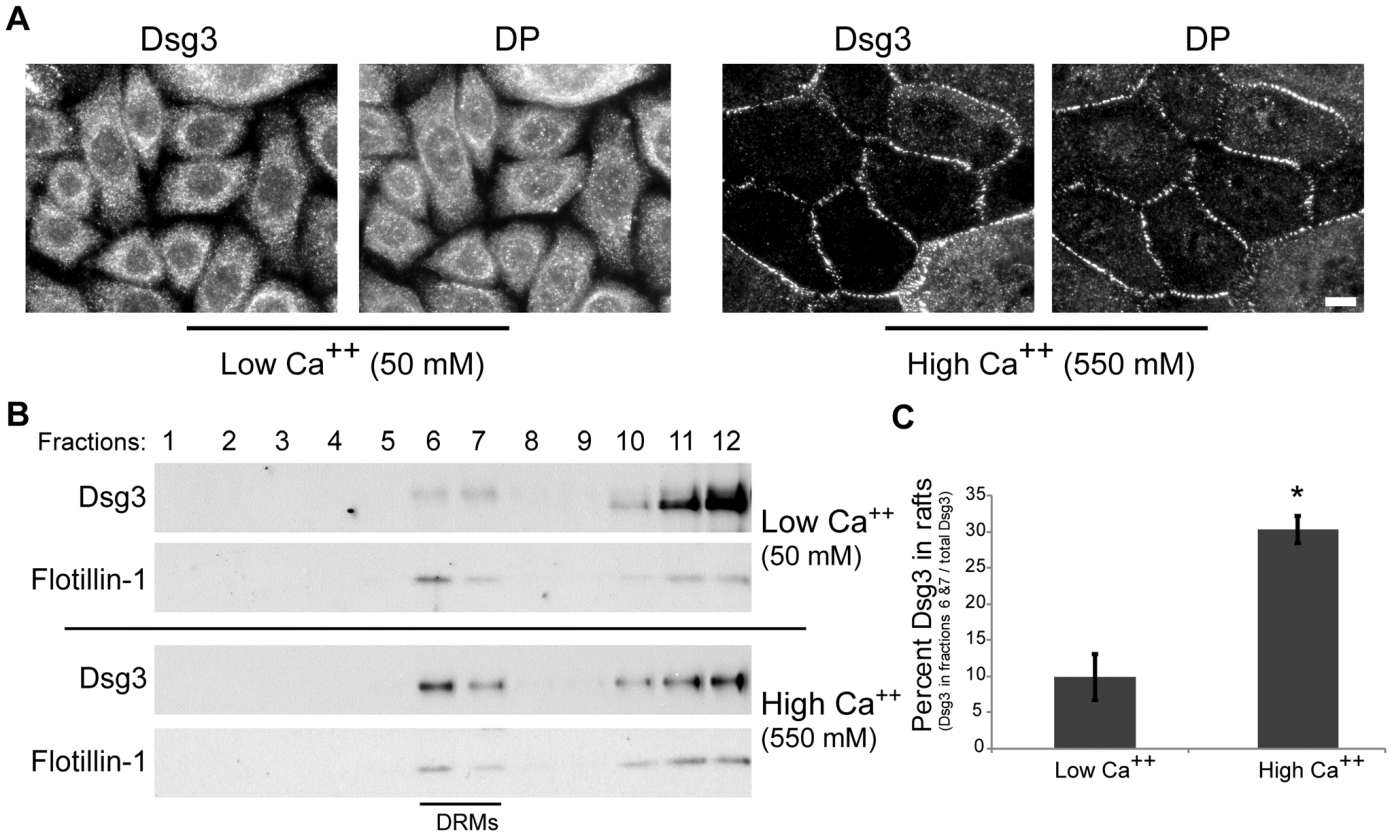
Dsg3 raft association increases upon calcium addition. (**A**) Dsg3 and DP (desmoplakin) remain cytoplasmic when human keratinocytes are cultured in low (50 µM) calcium media. Protein staining increases at regions of cell contact when keratinocytes are cultured in high (550 µM) calcium media and desmosomes assemble. Dsg3 was detected with AK23 mAb post fixation. (**B**) Confluent keratinocytes cultured in 50 µM or 550 µM calcium media 16–18 hrs prior to solubilization with 1% Triton X-100 and membrane raft fractionation. Western blots were probed for Dsg3 and the raft marker flotillin-1. Dsg3 raft partitioning increases significantly upon shifting cells from low to high calcium conditions. (**C**) Quantification of relative Dsg3 levels normalized to total Dsg3 across all fractions. Means ± SEM (*n* = 3); *p<0.05. Scale bar in A, 10 µm.

**Figure 5:**
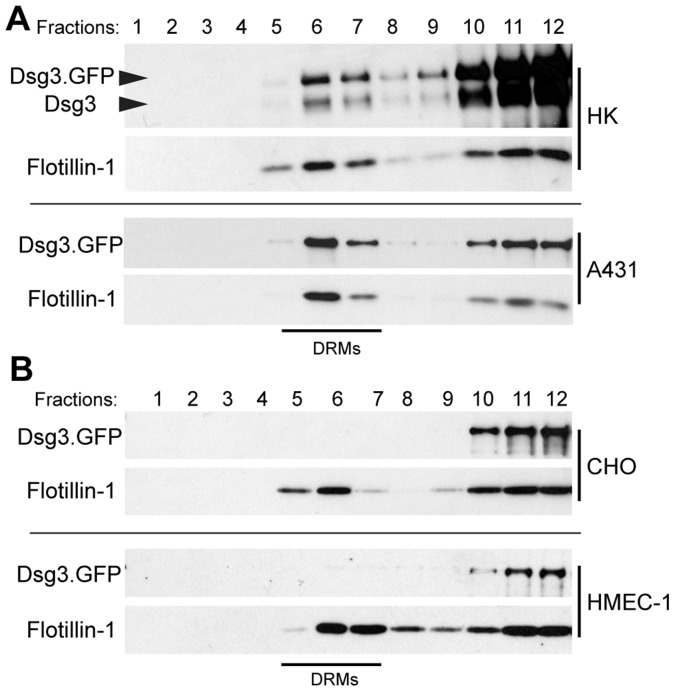
Dsg3 does not partition to rafts in cells lacking desmosomal proteins. (**A**) In human keratinocytes (HKs) cultured in high calcium media for 16–18 hrs and A431 cells, GFP-tagged Dsg3 (top arrow) partitions to rafts similar to endogenous Dsg3. (**B**) Dsg3.GFP was expressed in CHO (Chinese hamster ovary) cells and HMEC-1s (immortalized human microvasular endothelial cells), cell types that do not form desmosomes. Dsg3.GFP did not partition to the raft containing fractions in either CHOs or HMEC-1s.

A prediction derived from the results above is that only desmosomal Dsg3 is raft associated. To test this idea, raft association of Dsg3 was first compared between human keratinocytes cultured in low or high calcium medium. Keratinocytes cultured in low calcium medium do not form desmosomes as indicated by the cytoplasmic staining of both Dsg3 and desmoplakin (Fig. 4A). However, once exposed to high calcium, keratinocytes readily form desmosomes as indicated by the concentrated border staining of Dsg3 and desmoplakin (Fig. 4A). Interestingly, Dsg3 raft association increased significantly upon shifting cells from low to high calcium conditions (Fig. 4B, C). Dsg3 raft association was further analyzed in cell types with varying abilities to form desmosomes. For these experiments, wild type Dsg3.GFP was expressed in normal human keratinocytes and A431 cells, an epidermoid carcinoma cell line that forms desmosomes, or in CHO (Chinese hamster ovary) and HMEC-1 (immortalized human microvascular endothelial cells) cells, both of which lack various desmosomal components and therefore do not form desmosomes. As expected, Dsg3.GFP partitioned to rafts similarly to endogenous Dsg3 in primary HKs and showed substantial partitioning to rafts in A431s (Fig. 5A). However, Dsg3.GFP exhibited little or no raft association in CHOs and HMEC-1s (Fig. 5B), indicating that Dsg3 preferentially targets to raft fractions in a cell-type specific manner.

### PV IgG-induced Desmosome Disassembly is Raft-dependent

**Figure 6:**
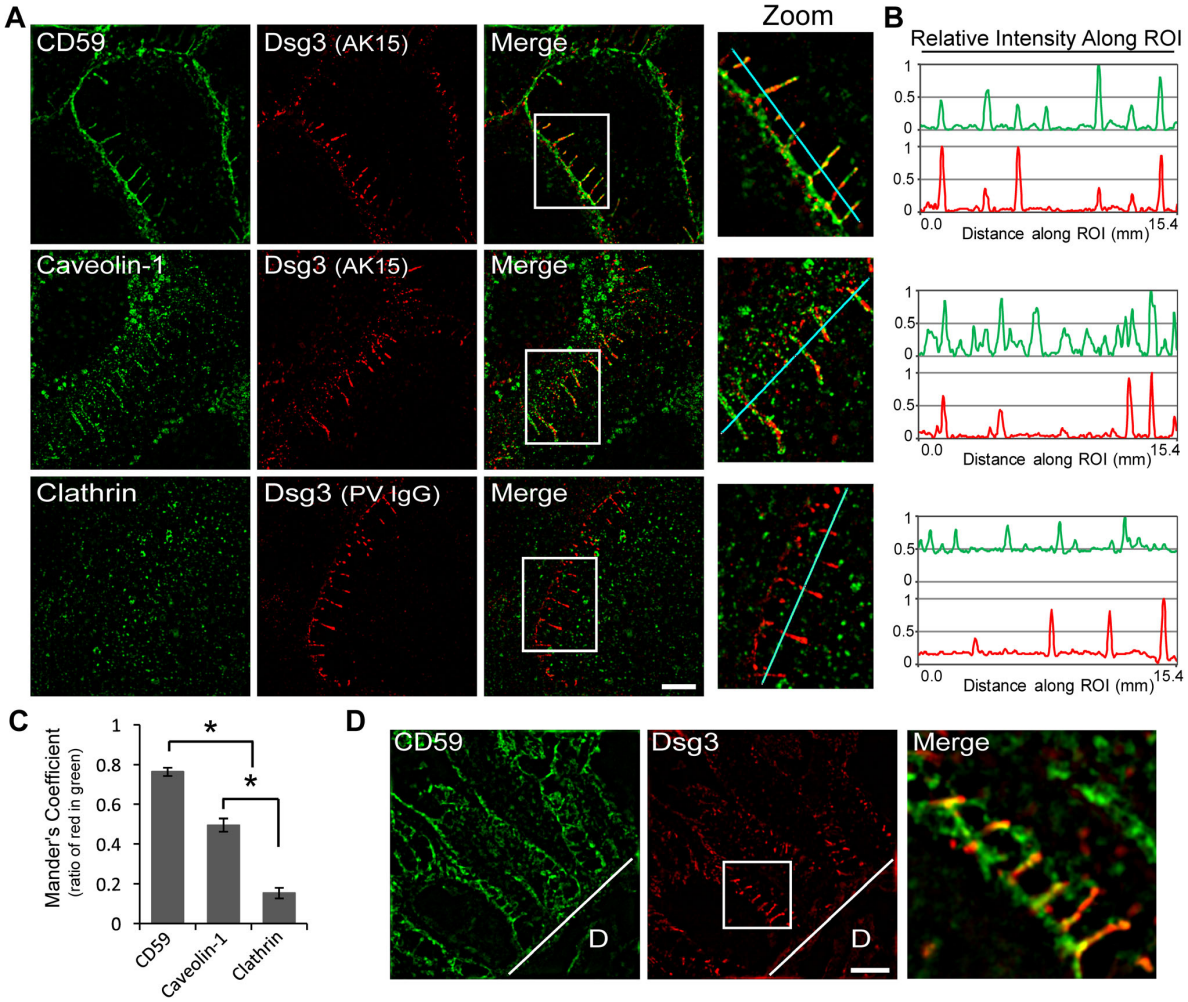
PV IgG causes redistribution of Dsg3 into raft-containing linear arrays. (**A–C**) Dsg3 colocalization with various membrane markers was analyzed using structured illumination microscopy (SIM) in human keratinocytes cultured in high calcium for 16–18 hrs and then treated with PV IgG for 3 hrs. CD59 was detected with FITC conjugated antibody by live labeling for 10 min prior to fixing. Dsg3 was detected with Alexa Fluor 555 conjugated AK15 mAb by live labeling for 10 min prior to fixing for the top two rows. For clathrin colocalization Dsg3 was monitored using PV IgG and secondary antibody detection of human IgG. In response to PV IgG, Dsg3 enters endocytic linear structures (previously termed ‘linear arrays’) that emanate perpendicular from cell-cell borders and extend toward the cell center (Jennings *et al.,* 2011). (**A**) Raft markers CD59 and caveolin-1 were enriched in linear arrays and colocalizaed significantly with Dsg3 relative to the non-raft marker clathrin. (**B**) Fluorescence intensity measurements of lines drawn perpendicularly through linear arrays show alignment of Dsg3 (bottom line) and raft marker (top line) fluorescence. (**C**) Quantification of Dsg3 colocalization in linear arrays as indicated by Mander’s coefficient (ratio of red in green). Means ± SEM (*n* = 27–36 arrays per group); *p<0.05. (**D**) SIM was also used to view Dsg3 colocalization with CD59 in linear arrays in excised normal human epidermis injected with PV IgG. Basal keratinocytes are shown. D, dermis. Scale bar in A and D, 5 µm.

Previous work from our laboratory has shown that Dsg3 endocytosis in response to PV IgG occurs via a raft-mediated pathway [23]. Recently, we have shown that PV IgG causes clustering and rearrangement of cell surface Dsg3 into endocytic linear structures that we have termed linear arrays. These structures extend perpendicularly from cell contacts and are sites for internalization of desmosomal components [32]. Given that endoyctic vesicles bud from these arrays and that internalization is raft-mediated, we predicted that raft components were enriched in these structures. Following PV IgG treatment, SIM revealed that raft markers CD59 and caveolin-1 were enriched in linear arrays, and colocalized significantly with Dsg3 relative to the non-raft marker clathrin in cultured human keratinocytes (Fig. 6A, C). Fluorescence intensity measurements of lines drawn perpendicularly through the linear arrays confirmed alignment of Dsg3 and raft markers (Fig. 6B). To determine if raft-enriched linear arrays also occur in human epidermis, excised human skin was injected with PV IgG and then analyzed by SIM (Fig. 6D). Dsg3, along with the raft marker CD59, was found in linear array structures remarkably similar to those observed *in vitro* (Fig. 6A). These results confirm enrichment of raft components in linear arrays, suggesting that linear array formation and subsequent desmosome disassembly are raft-dependent.

**Figure 7:**
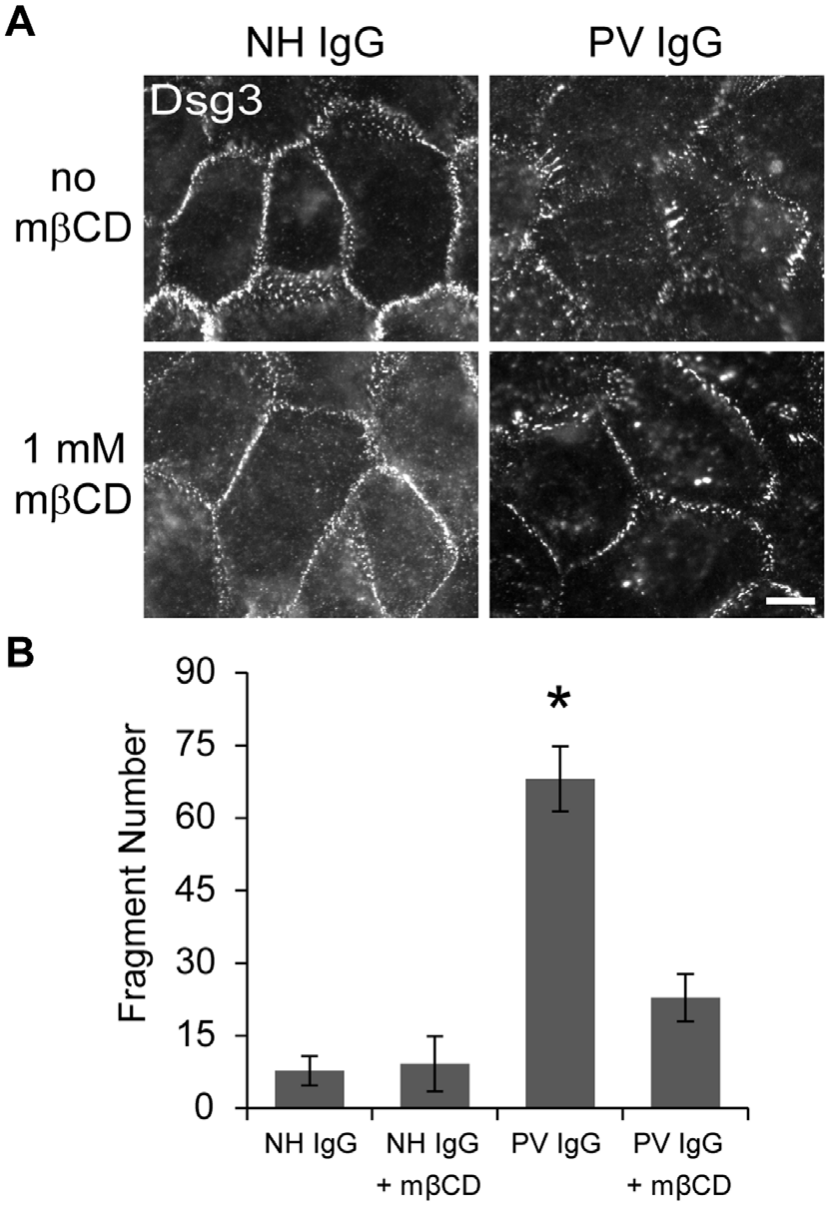
Cholesterol depletion prevents PV IgG-induced Dsg3 redistribution and weakened adhesion. (**A**) Human keratinocytes with assembled desmosomes were treated with NH or PV IgG for 3 hrs either in the absence or presence of 1 mM mβCD. Dsg3 was detected by live labeling with AK15 for 30 min on ice. Keratinocytes treated with PV IgG exhibit disrupted Dsg3 staining (surface clustering and linear array formation). mβCD treatment prevented both Dsg3 clustering and linear array formation in response to PV IgG. (**B**) mβCD treatment protected desmosomes against PV IgG-induced fragmentation. Means ± SEM (*n* = 4–8 monolayers per group); *p<0.05. Scale bar in A, 10 µm.

To test the possibility that PV IgG-induced linear array formation and loss of adhesion are raft-dependent, human keratinocytes were cultured in high calcium media to first allow for desmosome assembly, and subsequently treated with NH or PV IgG either in the absence or presence of mβCD. PV IgG disrupted Dsg3 staining as indicated by extensive cell surface clustering and linear array formation (Fig. 7A). These morphological changes were prevented by mβCD treatment, suggesting that PV IgG-induced desmosome disassembly was abrogated by cholesterol depletion. A fragmentation assay confirmed that desmosomes were functionally protected against PV IgG by mβCD (Fig. 7B). These results indicate that desmosome disassembly and loss of cell-cell adhesion in response to PV IgG require functional raft domains.

## Discussion

Significant advances have been made in characterizing desmosomes morphologically and biochemically [41]. A wide variety of human diseases have revealed critical functions of these adhesive junctions in the skin and heart [8], [12], [42]. However, we lack a comprehensive understanding of how desmosome assembly and function are regulated. The results presented here provide insight into how cells spatially control the dynamics of desmosome assembly and how these dynamics are altered to facilitate desmosome disassembly in diseases such as PV. Here, we demonstrate that desmosomal proteins associate with membrane rafts biochemically and that Dsg3 colocalizes with raft markers at cell borders. Dsg3 raft association increased during calcium-mediated desmosome assembly, and raft disruption by cholesterol depletion prevented both desmosome assembly and PV IgG-induced desmosome disassembly. These results support a model in which membrane raft microdomains serve as a crucial platform for desmosome regulation.

Several junctional proteins have been shown to be raft associated, including the tight junction proteins occludin, ZO-1 and JAM-A [43], [44] and the adherens junction protein N-cadherin [45], [46]. More recently, desmosomal proteins Dsg2, Dsc2, plakoglobin and desmoplakin also have been shown to be associated with membrane rafts [27], [28], [29], [47]. Here, we demonstrate for the first time that Dsg3 and other desmosomal proteins are raft associated in primary human keratinocytes (Fig. 1). The association of desmosomal proteins with rafts in cell types ranging from colonic and kidney epithelia to keratinocytes and carcinoma cell lines suggests that lipid raft modulation of desmosome function is a fundamental feature of desmosome regulation. Super-resolution imaging revealed that Dsg3 colocalized with raft markers CD59 and caveolin-1 at sites of cell-cell borders (Fig. 2), suggesting that rafts play a role in mediating desmosome assembly at regions of cell contact.

Current models for desmosome assembly suggest that desmosomal cadherins are stabilized in response to cell-cell contact and cluster with plaque proteins to yield a highly ordered and compact structure [4]. Our results showing that desmosome assembly is raft-dependent (Fig. 3) supports a model in which the raft milieu allows for and facilitates the extensive clustering that yields a mature and tightly packed desmosome. Even with a mβCD dose 10-fold lower than previously reported to weaken desmosomal adhesion [28], we found desmosome assembly and adhesion to be cholesterol dependent (Fig. 3). Importantly, Dsg3 was unable to associate with rafts in both CHO and HMEC-1 cells, suggesting that desmosomal or other proteins absent in these cells are responsible for Dsg3 raft targeting. Although Dsg3 colocalizes extensively with CD59 (Fig. 2), this protein does not appear sufficient for raft targeting of Dsg3 since HMEC-1 cells express this raft associating protein (Fig. S1). Collectively, these observations suggest that association of desmosomal proteins with raft domains plays an important role in desmosomal protein clustering and desmosome assembly. Raft dependent protein clustering has been demonstrated for both the immunological synapse and the neuromuscular junction [48], [49], [50], [51]. Evidence suggests that nanoscale rafts coalesce into larger and more stable membrane-ordered assemblies [25], [26], [52]. Desmosomes may represent a sub-type of these large raft-containing, stable membrane-ordered assemblies which result from the clustering of precursor pools associated with smaller nanoscale raft subcomplexes. Consistent with this possibility, early studies revealed that the lipid content of desmosome cores is enriched in cholesterol and sphingolipids [30], [31].

Our results investigating compromised adhesion in response to PV IgG also support a model in which raft-dependent clustering enables cadherin endocytosis and desmosome disassembly. We recently demonstrated that PV IgG cause extensive clustering of cell surface Dsg3, an effect that is attributed to the polyclonal nature of anti-Dsg3 IgG present in PV patients [19]. This combination of both loss of adhesion and clustering appears to drive Dsg3 endocytosis through a membrane raft pathway. Interestingly, polyclonal antibody-induced clustering of raft-localized proteins, such as placental alkaline phosphatase (PLAP), is cholesterol dependent [53]. The results in Figure 7 showing PV IgG-induced desmosome disassembly and loss of adhesion are cholesterol dependent, along with our work showing PV IgG cause clustering of cell surface Dsg3, suggest this antibody-induced clustering is raft-mediated. In addition to clustering of Dsg3, we previously demonstrated that PV IgG cause rearrangement of desmosomal proteins into linear array structures that function as sites for endocytosis [32]. Consistent with our study showing that PV IgG-induced internalization occurs via a raft-mediated pathway [23], super-resolution immunofluorescence imaging confirms that these linear array structures are enriched in raft markers (Fig. 6). Furthermore, raft-enriched linear arrays were also present in excised normal human skin treated with PV IgG (Fig. 6C). These findings suggest that drugs that alter raft dynamics may have therapeutic potential in treating PV.

**Figure 8:**
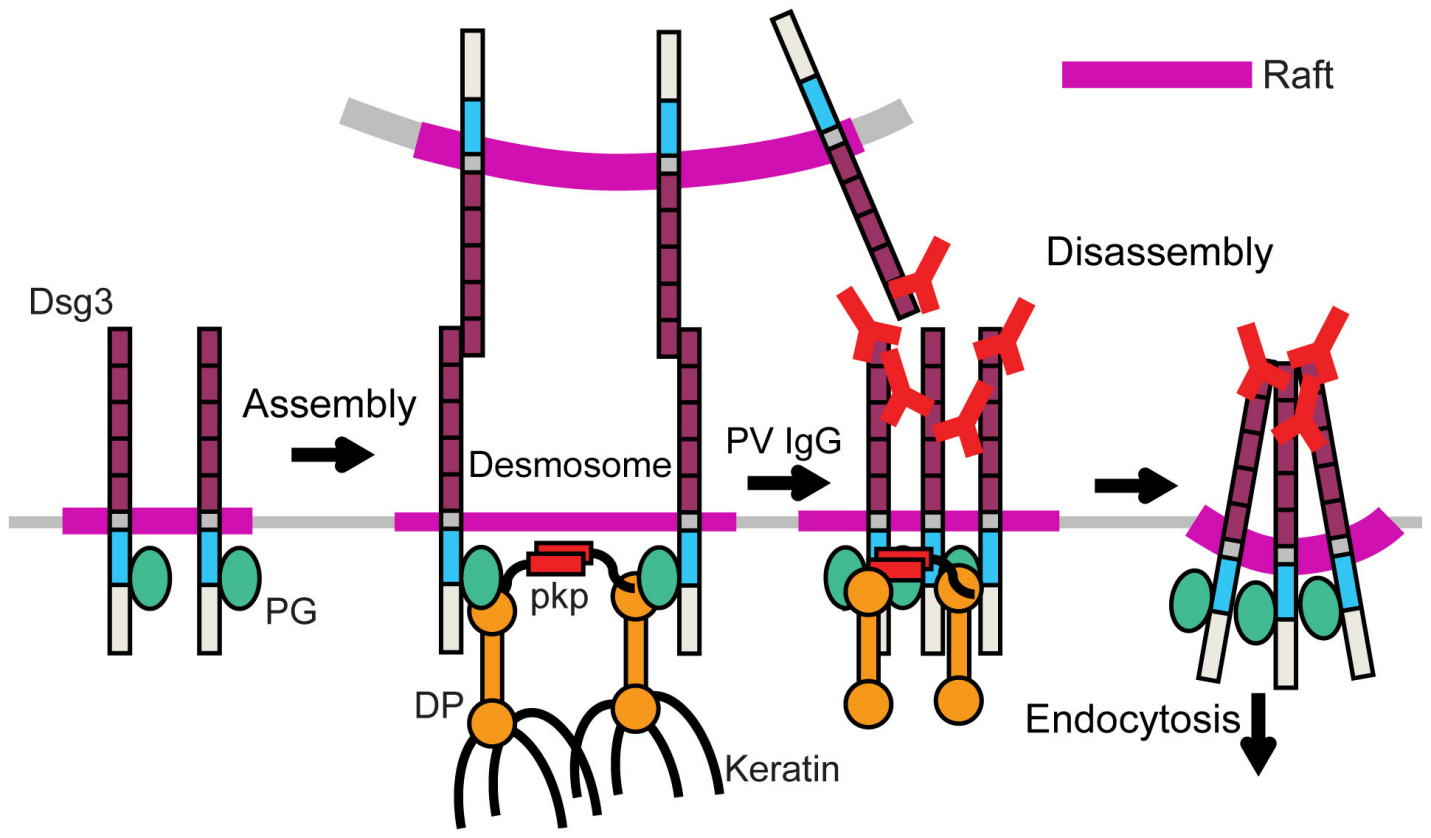
Model for membrane rafts as platforms for desmosome regulation. Desmosomal protein targeting to membrane rafts is required for the extensive clustering driven by cadherin ectodomain and plaque protein interactions during assembly that yields a mature and tightly packed desmosome. When adhesion is compromised (i.e. in response to PV IgG), clustering in a raft facilitates desmosomal cadherin endocytosis. PG, plakoglobin; pkp, plakophilin; DP, desmoplakin.

The results presented here support a model (Fig. 8) in which desmosomal protein association with ordered membrane raft domains is essential for clustering driven by cadherin ectodomain and plaque protein interactions during desmosome assembly, as well as for desmosomal cadherin clustering and endocytosis during disassembly in response to PV IgG. This model accounts for the somewhat counterintuitive notion that disruption of raft microdomains impacts both desmosome formation on the one hand, and desmosome disassembly in the context of PV on the other. In addition to functioning as platforms for desmosomal protein clustering during desmosome formation and disassembly, rafts may also function as signaling hubs for the recruitment of regulatory proteins that modulate desmosome formation and turnover. For example, desmosome disassembly in disease states such as PV has been linked to EGFR, p38MAPK and Src signaling [9], [14], [54], all of which have been found to be raft associated [38]. The ability of rafts to compartmentalize proteins at the membrane is a fundamental mechanism by which domains form platforms of specific composition for various cellular processes [26], [52], [55]. Thus, raft association is likely an efficient mechanism for compartmentalization of desmosomal components and their effectors (kinases, caspases, etc.) to precisely control desmosome assembly and disassembly dynamics. These findings further suggest that manipulation of raft dynamics may be a promising therapy to treat desmosomal diseases, such as pemphigus vulgaris and other disorders where desmosome function or signaling is compromised.

## Materials and Methods

### Ethics Statement

Use of human IgG and skin was approved by the Institutional Review Boards at Emory University and the University of Pennsylvania. This study used existing or otherwise discarded, de-identified human samples obtained for clinical purposes during medical evaluation or skin surgery repair procedures. These studies were approved for human subjects research exemption (informed consent not required) by the Institutional Review Board at the University of Pennsylvania and the Institutional Review Board at Emory University according to the United States Health and Human Services Code of Federal Regulations 46.101(b)(4).

### Cells and Culture Conditions

Human epidermal keratinocytes were isolated from neonatal foreskin as previously described [21] and cultured in KBM-Gold basal medium (Lonza, Walkersville, MD) with growth supplements (KGM-Gold Single-Quot Kit, Lonza). Keratinocytes, no later than passage 4, were switched to 550 µM calcium 16–18 hours prior to experimental manipulation, unless other noted. For the experiments in Figure 4 and Figure 8B, keratinocytes were grown in 50 µM calcium to prevent desmosome assembly for 16–18 hours and then switched to 550 µM calcium for the indicated times. Chinese hamster ovary (CHO) cells were cultured in F12 media (ATCC, Manassas, VA). HMEC-1s were cultured in 0.1% gelatin-coated flasks in EBM-2 media (Lonza) with growth supplements (EGM-2 MV SingleQuots, Lonza). Where indicated, cells were infected 24–48 hours prior to experimentation with adenovirus for expression of Dsg3.GFP, as previously described [32].

### Antibodies and Reagents

Antibodies used were as follows: mouse anti-Dsg3 antibodies AK15 and AK23 [33] were kind gifts from Dr. Masayuki Amagai (Keio University, Tokyo); mouse anti-Dsg3 antibody 5G11 (Invitrogen, Carlsbad, CA); mouse anti-plakophilin 2 antibody (Biodesign, Saco, Maine); rabbit anti-calnexin antibody (Enzo Life Sciences, Farmingdale, NY); mouse anti-CD59-FITC conjugated antibody (Millipore, Billerica, MA; Invitrogen); rabbit anti-desmoplakin antibody NW6 was a kind gift from Dr. Kathleen Green (Northwestern University); rabbit anti-gamma catenin antibody (plakoglobin, H-80, Santa Cruz Biotechnology, Santa Cruz, CA); mouse anti-E-cadherin antibody, mouse anti-flotillin-1, rabbit anti-caveolin-1, and mouse anti-clathrin antibodies (BD Biosciences, San Jose, CA). Secondary antibodies conjugated to Alexa Fluors were purchased from Invitrogen while horseradish peroxidase-conjugated secondary antibodies were purchased from BioRad (Hercules, CA). Normal human (NH) IgG was purchased from Bethyl Labs (Montgomery, TX). PV sera were kind gifts from Dr. M. Amagai, Dr. John Stanly (University of Pennsylvania, Philadelphia, PA) and Dr. Robert Swerlick (Emory University, Atlanta, GA). IgG was purified from PV sera using Melon Gel IgG Purification Resins and Kits (Thermo Fisher Scientific, Rockford, IL) according to the manufacturer’s protocol. methyl-β-cyclodextrin (mβCD) was purchased from Sigma (St. Louis, MO). 10 mM mβCD working stock was prepared by dissolving 15 mg in 1 mL pre-warmed keratinocyte culture medium. The solution was rotated for 30 min and subsequently sterile filtered (0.22 µm) prior to use.

### Isolation of Detergent Resistant Membranes

Detergent resistant membranes were isolated as described previously [34]. Briefly, cells were cultured in 25 cm^2^ flasks (two per gradient) and washed with PBS^+^. Cells were collected by scraping in TNE buffer supplemented with protease inhibitors (Roche Diagnostic GmbH) followed by centrifugation at 0.4 rcf at 4°C for 5 min (5415R, Eppendorf). Cells were re-suspended in TNE buffer and homogenized using a 25-guage needle. TNE buffer with detergent was added to lysate for a 1% Triton-X 100 final concentration followed by incubation on ice for 30 min. 400 µL was mixed with 800 µL 56% sucrose and placed at the bottom of a centrifuge tube. 1.9 mL volumes of 35% and 5% sucrose were layered on top of the sample. Following an 18 hour centrifugation at 4°C (44,000 rpm, SW55 rotor, Beckman Optima LE-80 K Ultracentrifuge), 420 µL fractions (1–11, remaining volume combined to make up fraction 12) were removed from top to bottom of the gradient and stored at −20°C until processed for western blot analysis. Flotillin-1 and calnexin were used as raft and non-raft markers respectively. Sucrose concentrations across gradients were measured using a AR200 Digital Refractometer (Leica).

### Differential Detergent Extraction

Keratinocytes were cultured until confluent in 4-well tissue culture plates. Cells were extracted sequentially in Triton buffer (1% Triton X-100, 10 mM Tris, pH 7.5, 140 mM NaCl, 5 mM EDTA, 2 mM EGTA, with protease inhibitor) followed by extraction with urea-SDS buffer (1% SDS, 8 M Urea, 10 mM Tris-HCl, pH 7.5, 5 mM EDTA, 2 mM EGTA) as described previously [21] and then processed for western blot analysis.

### Western Blot Analysis

Samples were mixed 1∶1 with Laemmli sample buffer containing β-mercaptoethanol and heated to 95°C for 5 min. Proteins were resolved by 7.5% SDS-PAGE and transferred to a nitrocellulose membrane according to standard protocols. Dsg3 was detected using an anti-Dsg3 antibody mixture (AK15 and 5G11). HRP-conjugated secondary antibodies were detected using enhanced chemiluminescence (GE Healthcare, Little Chalfont, Buckinghamshire, UK).

### Immunofluorescence

Human keratinocytes were cultured to 70% confluence on glass coverslips and then fixed on ice in either methanol for 2 min or 4% paraformaldehyde for 10 min followed by 0.2% Triton X-100 for 7 min. Primary antibodies described above were detected with Alexa Fluor-conjugated secondary antibodies. Wide field fluorescence microscopy was performed using a DMRXA2 microscope (Leica, Wetzler, Germany) equipped with a 63×/1.32 NA oil immersion objective and narrow band pass filters. Images were acquired with an ORCA digital camera (Hamamatsu Photonics, Bridgewater, NJ) and processed using Simple PCI software (Hamamatsu Corporation, Sewickley, PA). Super-resolution microscopy was performed using a Nikon N-SIM system on an Eclipse Ti-E microscope system equipped with a 100×/1.49 NA oil immersion objective, 488- and 561-nm solid-state lasers in 3D structured illumination microscopy mode. Images were captured using an EM charge-coupled device camera (DU-897, Andor Technology) and reconstructed using NIS-Elements software with the N-SIM module (version 3.22, Nikon). Colocalization analysis was performed by obtaining Mander’s coefficient using ImageJ plugin JACoP [35].

### Dispase-based Fragmentation Assay

Keratinocytes were cultured until confluent in 4-well tissue culture plates and processed as previously described [19]. Keratinocytes were switched to 50 µM calcium, to ensure no desmosome assembly, 16–18 hours prior to a second switch 550 µM calcium for 3 hours to assemble desmosomes. Where indicated, PV IgG (100 µg/mL) was added for another 3 hours at 37°C. Cells were then incubated with 1 U/mL dispase (Roche) for 30–45 min or until keratinocyte sheets were lifted from the culture dish. Cell sheets were rinsed with PBS^+^ and subjected to mechanical stress via pipetting. Cells were then fixed in paraformaldehyde, stained with methylene blue, and fragments counted using a dissecting microscope.

### Human Skin Explant Injections

Normal human skin explants were cultured as previously described [19]. PV IgG (160–400 µg) was injected intradermally in the presence of 0.8 µg ETA for 16 hours. Skin sections were processed for structured illumination immunofluorescence using a mouse anti-human CD59-FITC antibody and goat anti-human Alexa Fluor 555 secondary antibody.

### Statistics

Statistical analysis of fluorescence colocalization measurements in Figure 2 was performed using Kruskal-Wallis one-way analysis of variance on ranks with multiple comparisons performed by Dunn’s method with a significance level of 0.05. Statistical analysis of fluorescence colocalization measurements in Figure 6 and the dispase-based fragmentation assay in Figure 7 were performed using Shapiro-Wilk one-way analysis of variance on ranks with multiple comparisons performed by Holm-Sidak method with a significance level of 0.05. Statistical analysis of the dispase-based fragmentation assay in Figure 3 and raft association in Figure 4 were performed using a *t*-test assuming unequal variances with a significance level of 0.05.

## Supporting Information

Figure S1 **CD59 is expressed on the surface of HMEC-1 cells.** HMEC-1s were unlabeled (no antibody control) or labeled live with FITC-conjugated CD59 for 10 min at 37°C. The cells were then fixed in methanol and imaged. Labeling demonstrated that HMEC-1 cells express CD59. Negative control showed a lack of background fluorescence.
(TIF)
