# Supplementary figures and images for: Desmosome Assembly and Disassembly Are Membrane Raft-Dependent
Source: PLoS One. 2014 Jan 30;9(1):e87809. doi: 10.1371/journal.pone.0087809 (PMC3907498; doi:10.1371/journal.pone.0087809)

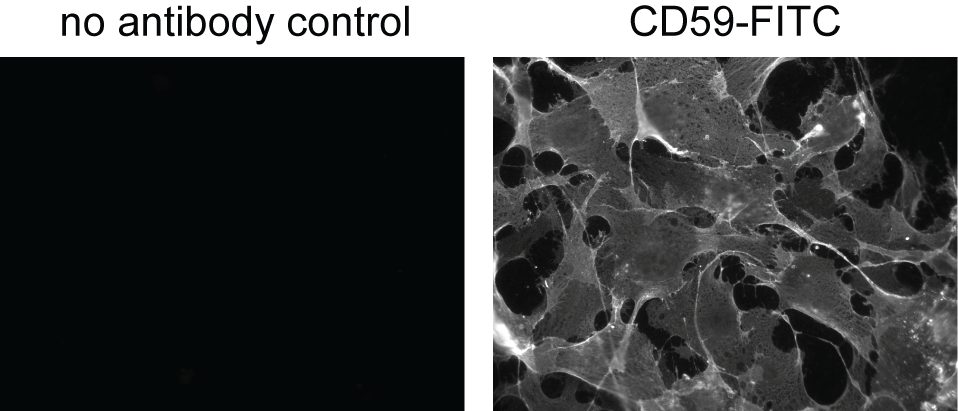

Supplement: Figure S1 — CD59 is expressed on the surface of HMEC-1 cells. HMEC-1s were unlabeled (no antibody control) or labeled live with FITC-conjugated CD59 for 10 min at 37°C. The cells were then fixed in methanol and imaged. Labeling demonstrated that HMEC-1 cells express CD59. Negative control showed a lack of background fluorescence. (TIF) [file pone.0087809.s001.tif]
